# O.2.3-11 Light standing work and high intensity occupational physical activities predict baseline and 8-year progression of carotid atherosclerosis among women

**DOI:** 10.1093/eurpub/ckad133.137

**Published:** 2023-09-11

**Authors:** Mette Korshøj, Karen Allesøe, Ole Steen Mortensen, Volkert Siersma, Jussi Kauhanen, Niklas Krause

**Affiliations:** Department of Occupational and Social Medicine, Holbæk Hospital, part of Copenhagen University Hospital, Denmark; Department of Occupational and Social Medicine, Holbæk Hospital, part of Copenhagen University Hospital, Denmark; Center for Clinical Research and Prevention, Copenhagen University Hospital - Bispebjerg and Frederiksberg, Copenhagen, Denmark; Department of Occupational and Social Medicine, Holbæk Hospital, part of Copenhagen University Hospital, Denmark; Section of Social Medicine, Department of Public Health, University of Copenhagen, Copenhagen, Denmark; The Research Unit for General Practice and Section of General Practice, Department of Public Health, University of Copenhagen, Copenhagen, Denmark; Institute of Public Health and Clinical Nutrition, University of Eastern Finland, Kuopio, Finland; Department of Epidemiology, University of California Los Angeles (UCLA), Los Angeles, CA, USA; Department of Environmental Health Sciences, University of California Los Angeles (UCLA), Los Angeles, CA, USA; melars@regionsjaelland.dk

## Abstract

**Purpose:**

Recent reviews link higher levels of occupational physical activity (OPA) to cardiovascular disease (CVD). However, the evidence for women is inconsistent and studies of activity-limiting symptomatic CVD are prone to healthy worker selection effect. To address these limitations, this study investigated OPA effects on asymptomatic carotid artery intima-media thickness (IMT), a proxy for atherosclerosis, among women.

**Methods:**

Participants include 905 women from the population-based Kuopio Ischemic Heart Disease Risk Factor Study with baseline (1998-2001) data on self-reported OPA and sonographic measurement of IMT. Linear mixed models with adjustment for 15 potential confounders estimated and compared mean baseline IMT and 8-year IMT progression for five levels of self-reported OPA. Analyses stratified by cardiovascular health and retirement status were planned because strong interactions between pre-existing CVD and OPA intensity have previously been reported.

**Results:**

Light standing work, moderately heavy active work, and heavy or very heavy physical work were all consistently associated with greater baseline IMT and 8-year IMT progression than light sitting work, being hazardous for cardiovascular health. The greatest baseline IMT was observed for heavy or very heavy physical work (1.21 mm), and the greatest 8-year IMT progression for light standing work and moderately heavy active work (both 0.13 mm), 30% above sitting work (0.10 mm). Stratified analyses showed that these differences were driven by much stronger OPA effects among women with baseline carotid artery stenosis. Retired women experienced slower IMT progression than those still working at baseline.

**Conclusions:**

Light standing work and higher levels of OPA intensity predict higher baseline IMT and 8-year IMT progression, especially among women with baseline stenosis. This study suggests that inconsistent findings regarding the effects of OPA on cardiovascular diseaes and mortality outcomes may be due to healthy worker selection and exposure misclassification biases including the failure to distinguish between standing and sitting work postures in many cohort studies.

**Funding Source:**

This project is funded by the Danish taxpayers, via the Danish Work Environment Research Foundation, grant number 20195100247.

